# Target detection increases pupil diameter and enhances memory for background scenes during multi-tasking

**DOI:** 10.1038/s41598-019-41658-4

**Published:** 2019-03-27

**Authors:** Khena M. Swallow, Yuhong V. Jiang, Elizabeth B. Riley

**Affiliations:** 1 Department of Psychology, Cornell University, Ithaca, NY USA; 2 Department of Psychology, University of Minnesota, Minneapolis, MN USA; 3 Department of Human Development, Cornell University, Ithaca, NY USA

## Abstract

Attending to targets in a detection task can facilitate memory for concurrently presented information, a phenomenon known as the *attentional boost effect*. One account of the attentional boost suggests that it reflects the temporal selection of behaviorally relevant moments, broadly facilitating the processing of information encountered at these times. Because pupil diameter increases when orienting to behaviorally relevant events and is positively correlated with increases in gain and activity in the locus coeruleus (a purported neurophysiological mechanism for temporal selection), we tested whether the attentional boost effect is accompanied by an increase in pupil diameter. Participants memorized a series of individually presented scenes. Whenever a scene appeared, a high or low pitched tone was played, and participants counted (and later reported) the number of tones in the pre-specified, target pitch. Target detection enhanced later memory for concurrently presented scenes. It was accompanied by a larger pupil response than was distractor rejection, and this effect was more pronounced for subsequently remembered rather than forgotten scenes. Thus, conditions that produce the attentional boost effect may also elicit phasic changes in neural gain and locus coeruleus activity.

## Introduction

Dividing attention across multiple tasks and stimuli typically results in poorer task performance^1^. These interference effects are increased when an item that requires an overt or covert response appears for one of the tasks^1^. A well-known example is the attentional blink, in which participants’ ability to report a target item (e.g., which character appeared in a red font) is impaired if it appears after another item that also requires a response. Deficits are also observed when two targets are presented at the same time, such as in the dual-target cost^2^. However, recent research has demonstrated that the opposite effect can also occur. In the *attentional boost effect* images that appear at the same time as an unrelated target for one task are later better remembered than images that coincide with other, nontarget, items^3–5^. Although the demands of the detection task are higher for detecting and responding to a target than for rejecting a distractor^2,6^, target detection facilitates both short- and long-term memory for concurrently presented images^3,4,7,8^. Evidence that target detection facilitates the processing of concurrently presented items is also found in perceptual priming^9^, when the stimuli are presented within the same or in different modalities^10^, and in memory for task-irrelevant information about the event^5,8,11^. Yet, current understanding of the mechanisms that produce the attentional boost effect is limited. This study uses pupillometry to further characterize the mechanisms involved in the attentional boost effect.

Current accounts of the attentional boost effect suggest that it reflects changes in attention during the initial encoding of an image into memory, rather than subsequent elaborative processing, consolidation, or retrieval. For example, factors that increase attention to a word or image (e.g., duration, orthographic distinctiveness) reduce the magnitude of the attentional boost effect^10,12,13^, implying that these manipulations engage overlapping mechanisms^10^. The effects are unlike those associated with spatial selection or distinctiveness, however^8,14,15^. The attentional boost effect occurs when the detection and encoding stimuli appear in separate spatial locations^7,10,16^, when perceptual load is high^4,8^, and when participants are instructed to ignore the background image^8^. In addition, the categorization of a stimulus as an item that requires an overt or covert response, rather than oddball or distinctiveness processing, is critical for the attentional boost effect^8^. The effect occurs when targets are as frequent as distractors, and when they are relatively rare^17^. However, because the attentional boost effect does not occur when the target precedes or trails the image by 100 ms^18^, it is unlikely to result from the fact that some images predict the presence of the target, or vice versa.

Based on these data, the *dual-task interaction* account of the attentional boost effect proposes that the categorization of an item as a target triggers a temporal selection mechanism that briefly, but broadly enhances the processing of information present during behaviorally relevant moments^14^. Consistent with this account, detecting a target in a time series elicits activity in the ventral attention network (including the right temporoparietal junction, middle frontal gyrus, and ventral frontal cortex), which is involved in interrupting processing and reorienting attention to salient events^19,20^. Target detection also produces transient increases in activity in visual and auditory regions, even when no stimuli from that modality are presented^6,21^. Thus, temporal selection could produce the attentional boost effect by broadly enhancing the processing of information presented at behaviorally relevant moments.

This study tested the feasibility of this account by examining differences in pupil responses to target and distractor trials during the encoding task. Temporal selection shares features with the orienting response associated with the onset of motivationally significant stimuli^19,22–25^. Though the orienting response likely includes multiple systems^26,27^ one potential neurophysiological mechanism underlying both temporal selection and the orienting response is phasic activity in the locus coeruleus (LC) system^14,23,24,28,29^. Whereas tonic levels of LC activity regulate wakefulness, arousal, and task engagement^28,30–32^, phasic increases in LC activity are involved in responding to behaviorally relevant stimuli, such as targets, and in adapting to changes in the current situation^33–36^. Growing evidence suggests that increases in LC activity are indexed by increases in pupil diameter^30,37,38^. One study that combined electrophysiology and microstimulation techniques provided evidence that changes in LC spiking rate (and other areas, including the inferior and superior colliculus^25^) produce phasic changes in pupil dilation in non-human primates^38^. Another recent study in mice demonstrated that phasic pupil responses to auditory tones are suppressed when LC neurons are silenced, suggesting a direct causal role in regulating pupil size^39^. In humans, tonic levels of pupil diameter vary with levels of sustained attention^31,32,40^. In addition, salient, surprising, or goal-relevant task events, such as a target or auditory tone, often elicit transient increases in pupil diameter (*phasic pupil response*^30,41–44^).

Though the literature provides many reasons to expect pupil diameter to increase more following targets than distractors in attentional boost effect tasks, it was necessary to test whether this is the case for several reasons. The attentional boost effect occurs when targets and distractors are equally frequent, when no overt response is made to the targets, and when detection task items are presented at short, regular intervals over extended periods of time (these conditions are not necessary^7^). In many investigations of phasic pupil and LC responses, the events used to elicit them are infrequent relative to other items, occur sporadically, or violate expectations^27,37,38,41,44–47^. The standard attentional boost paradigm also requires participants to simultaneously and continuously attend to multiple tasks and stimuli over extended periods of time, which could increase baseline levels of pupil diameter^48,49^ and limit phasic responses to targets^28,32,47,50^. Finally, the attentional boost effect is often performed with images that vary in luminance. Because attending to brighter scenes can increase pupil diameter^51–53^, testing whether the pupil response to targets depends on scene luminance will also be important.

 Investigating pupil responses to targets and distractors during encoding may also help link these data to several previous reports that have tied changes in pupil diameter during encoding to better subsequent memory. During effortful encoding, larger pupil responses to an item have been associated with greater likelihood of later recognizing it^27^ with high confidence^54^. However, the successful encoding of an item has also been associated with more rapid decreases in pupil diameter during an encoding task^55,56^. Thus, pupil diameter may reveal differences in the way items are processed during encoding. Examining whether these effects are modulated by the interaction between target detection and subsequent memory of concurrently presented items may therefore provide additional insight into the mechanisms that produce the attentional boost effect.

### The Current Study

**Figure 1:**
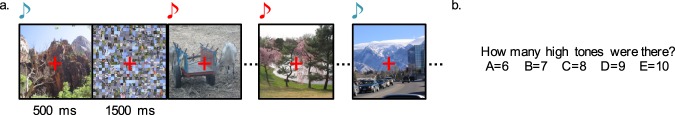
Encoding and counting task. (**a**) For the encoding and counting task, one tone (high or low frequency) was presented for 100 ms at the beginning of each trial. A scene was also presented at the center of the screen for 500 ms. Scenes were masked for 1500 ms (illustrated only for the first scene). There was no interval between trials. A red fixation cross appeared in the center of the screen throughout the task. Participants were instructed to keep their eyes on the fixation cross throughout the task. (**b**) The task paused every 6–10 targets so participants could report their count by pressing one of five keys (A–E). Sizes are not to scale.

This study examined differences in pupil responses to targets and distractors in the attentional boost effect task. Participants performed two tasks at the same time while their pupil diameter was recorded. For one task, participants intentionally encoded scenes that were individually, but briefly, presented in the center of the screen in a continuous series (Fig. 1a). Each scene was presented with a high or low pitched tone, one of which was assigned as the target tone. Half of the scenes were presented with the target tone, which participants counted to avoid any potential contributions of a motor response to pupil responses on target trials. The other half of the scenes were presented with a distractor tone, which required no response. Counting accuracy was assessed at irregular intervals throughout the task (Fig. 1b). After completion of the encoding and counting task, scene memory was tested in a two-alternative forced choice recognition test. For this test, participants were shown two scenes, and they had to select the scene that was shown to them in the initial encoding task. The attentional boost effect suggests that scenes that were presented at the same time as a target tone should be better remembered than those presented with a distractor tone. If the attentional boost effect results from temporal selection, then pupil diameter should increase more following a target than following a distractor. These differences may also be larger for target-paired scenes that are later remembered, relative to those that are later forgotten.

## Results

### Counting Task

Performance on the counting task was good, with a mean proportion of correct responses of 0.885 (SD = 0.077). When the response was incorrect, it was typically off by 1 in either direction (absolute difference when wrong M = 1.079, SD = 0.151).

### Recognition Memory

**Figure 2:**
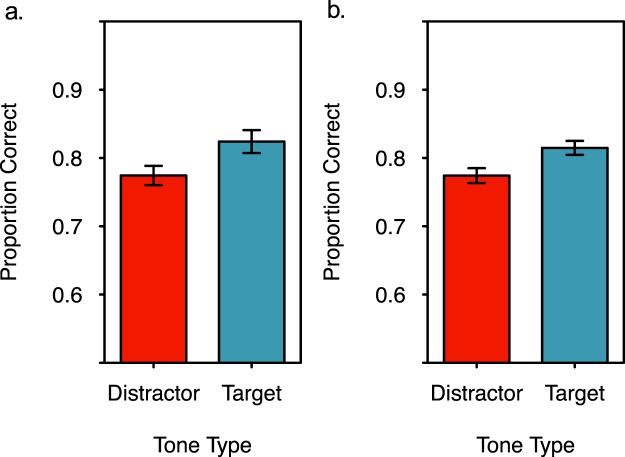
Recognition accuracy for scenes presented at the same time as target and distractor tones during encoding. (**a**) Individuals better recognized scenes that were presented at the same time as a target tone that they counted, than those presented at the same time as a distractor tone that was not counted (subject-level analysis). (**b**) Scenes were better recognized when they were presented at the same time as a target tone rather than a distractor tone (item-level analysis). Error bars represent +/− one standard error of the mean.

Despite increased demands on attention from the counting task, participants better recognized scenes that appeared with target tones during encoding than those that appeared with distractor tones (Fig. 2a). A paired samples, two tailed t-test indicated that this difference was significant, *t*(41) = 3.754, *p* = 0.001, *d* = 0.505. (For this and all subsequent statistical tests, α = 0.05 unless noted. In addition, the central limit theorem indicates that the sampling distribution of the mean for a sample this large should approach a normal distribution^57^).

Although these data replicated the attentional boost effect, the effect size was at the smaller end of the range that is typically reported for this type of task (6–20%^4,5^), with a mean difference between targets and distractors of 5%. This may reflect the longer trial duration relative to more typical approaches, as longer trial durations reduce the magnitude of the effect^12^.

To evaluate whether the attentional boost effect is likely to be observed with another sample of scenes^58^ an item-level analysis was performed on the recognition data^11^. Recognition data were averaged within scenes rather than within participants. After excluding scenes that were not in both the target and distractor conditions for at least five participants, this analysis had a sample size of 214 scenes. Results indicated that when a scene was presented at the same time as a target tone during encoding, it was later better recognized than when it was presented at the same time as a distractor tone during encoding, paired samples, two tailed t-test *t*(213) = 3.591, *p* < 0.001, *d* = 0.273 (Fig. 2b).

### Pupillary responses

**Figure 3:**
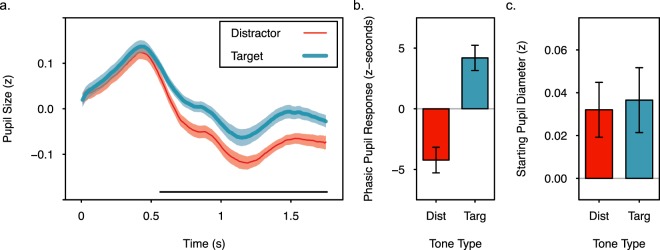
Pupil diameter increased following the onset of target and distractor tones, but was elevated longer following a target tone. (**a**) Standardized pupil diameter for each sample following the onset of a trial during the encoding and counting task. The black horizontal line indicates significant differences at *p* < 0.05 without correction for multiple comparisons (tests were performed every 83.3 ms, see text for exact times). (**b**) Phasic pupil response (area under the curve) for trials following target and distractor tones. (**c**) Starting pupil diameter during the first 100 ms following trial onset. Error bars and shading represent +/− one standard error of the mean.

Immediately following the presentation of a tone the pupil rapidly increased and then decreased in size (Fig. 3a). However, pupil diameter did not decrease as much for target tones as it did for distractor tones, and was relatively larger through the end of the target trials. This difference resulted in greater phasic pupil responses (area under the curve, see methods) for target trials than for distractor trials (Fig. 3b), paired samples, two tailed t-test, *t*(41) = 4.059, *p* < 0.001, *d* = 1.238. There were no significant differences in starting pupil diameter between target and distractor trials (Fig. 3c), paired samples, two tailed t-test, *t*(41) = 0.373, *p* = 0.711. To further characterize differences in the pupil responses on target and distractor trials, paired samples, two tailed t-tests were performed on every 10^th^ sample (83 ms intervals) after trial onset. With a Bonferroni corrected α level of 0.002, pupil diameter following target and distractor tones diverged approximately 0.667 s after tone onset and remained significantly different 1.667 s into the trial, all *t’s*(41) > 3.256, *p’s* < 0.002, *d’s* > 0.467. Without Bonferroni correction, pupil diameter was significantly different from 0.583 to 1.750 s, *t’s*(41) > 2.578, *p’s* < 0.014, *d’s* > 0.336.

**Figure 4:**
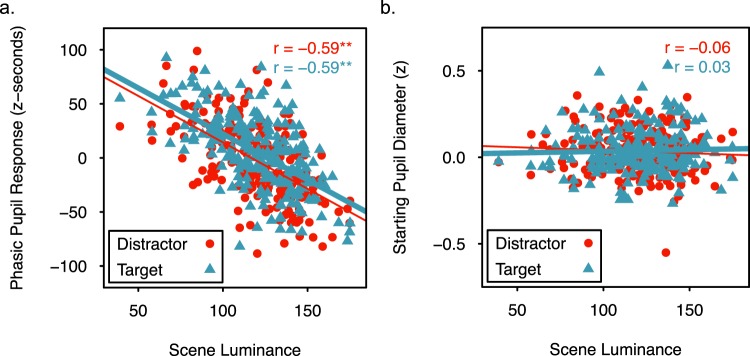
Relationship between scene luminance (range 0–255, see methods) and pupil diameter. (**a**) Phasic pupil responses (z-seconds) for scenes of varying luminance when paired with a target or distractor tone. (**b**) Starting pupil diameter (z) for scenes of luminance when paired with a target or distractor tone. For both panels, separate regression lines are included for target- and distractor-paired scenes. The r values indicate the Pearson correlation between the pupil measures and scene luminance in the target and distractor conditions. ** indicates correlations that are significant at p < 0.01 with 214 degrees of freedom.

Though phasic pupil responses should be modulated by scene luminance, it is possible that scene luminance influences the magnitude of the effects of encoding condition on pupil responses^51,53^. Therefore, a model comparison approach evaluated the effects of scene luminance (i.e., the mean grayscale pixel value of the scene, see methods) and encoding condition on phasic pupil responses to the individual scenes. For this item-level analysis, the phasic pupil response to a scene was averaged across participants and within condition. Three linear mixed effects models were then fit to the data and compared using lme4 and lmerTest in R^59,60^. The simplest model (*luminance only*) included scene luminance as a fixed effect and a random intercept for scene. The other two models additionally included either a main effect of encoding condition (*luminance* + *encoding condition*) or the main effect and interaction of encoding condition and luminance (*luminance × encoding condition*). Analysis of variance indicated that adding encoding condition to the models significantly increased the amount of explained variance in phasic pupil responses, but adding the interaction between luminance and encoding condition did not (Table 1). Thus, scene luminance and encoding condition, but not their interaction, influenced phasic pupil responses. Two tailed t-tests with Satterthwaite corrected degrees of freedom on the luminance + encoding condition model parameters indicated a significant negative relationship between scene luminance and phasic pupil responses, *ß* = −0.854, SE = 0.065, *t*(214) = −13.2, *p* < 0.001, and significantly greater phasic pupil responses following targets than distractors, *ß* = 7.962, SE = 2.243, *t*(213.99) = 3.55, *p* < 0.001. Both main effects are evident in Fig. 4a, which shows a decrease in the phasic pupil response as luminance increases, and an upward shift in the regression line when scenes are paired with a target rather than a distractor. These analyses confirm that pupil diameter increases following the presentation of a target in tasks that produce the attentional boost effect, and that this effect occurs across items that vary in scene luminance. This finding suggests that phasic pupil responses to targets may be independent of mechanisms that modulate pupil responses according to the luminance of covertly attended or imagined stimuli^51,53^.

**Table 1:** Comparison of linear mixed effects models of the phasic pupil response to evaluate the contributions of luminance, encoding condition (target vs. distractor), and their interaction.

Fixed Effects	AIC	Log Likelihood	df	*χ* ^2^	*p*
luminance only	4064.2	80.892	4		
luminance + encoding condition	4053.9	82.834	5	12.244	<0.001
luminance × encoding condition	4055.9	83.867	6	0.009	0.925

Note: χ^2^ tests compared models that differed in one parameter, and therefore had 1 df.

The same approach was used to verify that starting pupil diameter did not differ across conditions or scene luminance. The *luminance only* linear mixed effects model indicated that scene luminance did not account for a significant amount of variance in starting pupil diameter, *ß* < 0.001, SE < 0.001, *t*(214) = 0.308, *p* = 0.759. Including the main effect of encoding condition or the main effect and its interaction with scene luminance did not account for significantly more variance (Table 2). Thus, there were no significant effects of scene luminance or encoding condition on starting pupil diameter (Fig. 4b).

**Table 2:** Comparison of linear mixed effects models of starting pupil diameter to evaluate the contributions of luminance, encoding condition (target vs. distractor), and their interaction.

Fixed Effects	AIC	Log Likelihood	df	*χ* ^2^	*p*
luminance only	−540.48	274.24	4		
luminance + encoding condition	−538.61	274.31	5	0.131	0.718
luminance × encoding condition	−537.67	274.84	6	1.061	0.303

Note: χ^2^ tests compared models that differed in one parameter, and therefore had 1 df.

### Subsequent Memory Analyses

**Figure 5:**
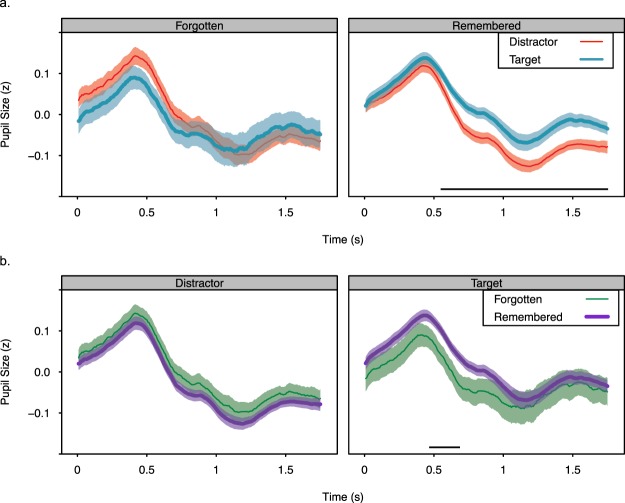
Standardized pupil diameter for each sample following the presentation of distractor-paired and target-paired scenes that were subsequently remembered or forgotten. The data are presented with two different groupings to facilitate comparison across conditions. In (**a**) the time courses for forgotten (left) and remembered (right) conditions are presented in separate plots. In (**b**) the time courses for distractor (left) and target (right) conditions are presented in separate plots. The horizontal lines indicate significant differences at *p* < 0.05, without correction for multiple comparisons (tests were performed every 83.3 ms, see text for exact times). Data are from the encoding phase. Shading represents +/− one standard error of the mean.

To examine whether larger phasic pupil responses to target tones than distractor tones is related to the attentional boost effect in memory, target and distractor trials were sorted based on whether the scene was later recognized correctly (*remembered*) or not (*forgotten*). The four resulting time-courses are illustrated in Fig. 5. Because recognition accuracy was high (~80%), there were fewer data points in the forgotten conditions (Table 3). This resulted in greater variance for forgotten scenes than for remembered scenes and decreased the power of the analyses. Nevertheless, analyses of these data provide some information about whether pupil responses differed when target-paired and distractor-paired scenes were subsequently remembered rather than forgotten. Analyses focused on measures that captured the phasic pupil response (defined as area under the curve), differences in pupil diameter throughout the trial, and the rate at which pupil diameter decreased following the initial peak (Tables 4–6). Unless otherwise indicated, these measures were analyzed with linear mixed effects models (lme4, lmerTest^59,60^) that included encoding condition, subsequent memory, and their interaction as fixed effects. Random intercepts and slopes for the effects of encoding condition and subsequent memory were included for each participant. Analysis of variance on these models, with Type III sums of squares and Satterthwaite corrected degrees of freedom, were performed.

**Table 3:** Number of observations in each condition defined by subsequently remembered and forgotten target- and distractor-paired scenes.

Subsequent Memory	Distractor				Target			
	Min	Max	Mean	SD	Min	Max	Mean	SD
Forgotten	2	22	12.605	5.165	1	25	9.86	5.978
Remembered	34	54	43.395	5.165	31	55	46.14	5.978

**Table 4:** Means and standard deviations (in parentheses) of pupil indices on trials including subsequently remembered and forgotten target- and distractor-paired scenes.

Condition	Phasic Pupil Response (z-seconds)	Slope (z/sec × 10e-3)	Starting Pupil Diameter (z)	Intercept (z)
Distractor				
Forgotten	−0.568 (19.968)	−2.775 (2.793)	0.046 (0.129)	0.259 (0.238)
Remembered	−5.026 (8.171)	−2.867 (2.601)	0.031 (0.097)	0.241 (0.213)
Target				
Forgotten	−3.526 (37.942)	−2.247 (3.504)	0.001 (0.200)	0.180 (0.286)
Remembered	4.146 (9.630)	−2.256 (2.535)	0.040 (0.099)	0.233 (0.199)

**Table 5:** Mean and standard deviation (in parentheses) of the parameters describing the post-peak decrease in pupil diameter.

Condition	Max Time (s)	Min Time (s)	Max Value (z)	Min Value (z)	Intercept (z)	Slope (z/sec × 10e-3)
Distractor						
Forgotten	0.446 (0.159)	1.251 (0.349)	0.182 (0.127)	−0.182 (0.141)	0.367 (0.285)	−3.976 (3.238)
Remembered	0.449 (0.163)	1.235 (0.325)	0.142 (0.101)	−0.181 (0.090)	0.338 (0.248)	−3.956 (2.974)
Target						
Forgotten	0.508 (0.260)	1.262 (0.366)	0.150 (0.194)	−0.177 (0.241)	0.321 (0.349)	−3.624 (3.548)
Remembered	0.516 (0.214)	1.290 (0.354)	0.167 (0.083)	−0.130 (0.111)	0.327 (0.275)	−3.209 (2.740)

**Table 6:** ANOVA tables for parameters used to describe the post-peak decrease in pupil diameter.

Statistic	Effect	SS	F	df	p
Max Time	Encoding Condition	1289.85	4.888	42.974	0.032
	Subsequent Memory	16.93	0.064	67.762	0.801
	Interaction	3.15	0.012	82	0.913
Min Time	Encoding Condition	229.844	0.577	41	0.452
	Subsequent Memory	10.846	0.027	41	0.870
	Interaction	298.667	0.750	41	0.392
Max Value	Encoding Condition	3.91e-4	0.041	49.301	0.840
	Subsequent Memory	3.746e-3	0.395	49.179	0.533
	Interaction	3.454e-2	3.639	82	0.060
Min Value	Encoding Condition	2.534e-2	1.779	53.442	0.188
	Subsequent Memory	1.273e-2	0.894	45.697	0.349
	Interaction	2.101e-2	1.475	82	0.228
Intercept	Encoding Condition	1.583e-2	0.567	41.226	0.456
	Subsequent Memory	4.678e-3	0.168	60.923	0.684
	Interaction	1.254e-2	0.449	82	0.505
Slope	Encoding Condition	4.619e-6	1.955	41	0.170
	Subsequent Memory	1.538e-6	0.651	41	0.425
	Interaction	1.649e-6	0.698	41	0.408

Note: Values listed for df are denominator degrees of freedom obtained with Satterthwaite’s method using lmerTest. Numerator degrees of freedom were 1 for all tests.

If the larger phasic pupil response to target tones than distractor tones is related to the attentional boost effect in memory, then subsequently remembered target-paired scenes may be associated with larger phasic pupil responses during encoding than those that are subsequently forgotten. This difference should be greater for target-paired scenes than for distractor-paired scenes, which could also show subsequent memory effects in pupil responses^27,54–56^. Consistent with this prediction, analyses of phasic pupil responses (area under the curve) revealed a significant interaction between encoding condition and subsequent memory, *F*(1, 82) = 5.144, *p* = 0.026. The two main effects did not reach significance, *F’s*(1, 41.349) < 0.887, *p*’s > 0.352. However, post-hoc comparisons with two-tailed, paired samples t-tests indicated that phasic pupil responses significantly differed for remembered target-paired vs. remembered distractor-paired scenes, *t*(41) = 3.818, *p* < 0.001, *d* = 1.027, but not for the other comparisons, *t’s*(41) < 1.242, *p’s* > 0.221. These analyses were repeated after accounting for differences in the luminance of the images. Residual pupil diameter was obtained from a linear model of pupil diameter with scene luminance, time, and their interaction as factors. Phasic pupil responses (area under the curve) were then recalculated. These analyses yielded the same results as the analysis on the non-residualized diameters: The interaction between encoding condition and subsequent memory was significant, *F*(1, 82) = 4.016, *p* = 0.048, but the two main effects were not, *F’s*(1, 41.413) < 1.107, *p*’s > 0.299. Post-hoc analyses again indicated that pupil responses significantly differed for remembered target-paired vs. distractor paired scenes, *t*(41) = 3.816, *p* < 0.001, *d* = 1.008, but not the other conditions, *t’s*(41) < 1.282, *p’s* > 0.207.

The interaction between encoding condition and subsequent memory was further characterized by testing when pupil diameter differed across the four conditions. Paired samples, two tailed t-tests comparing pupil diameter were separately performed on every 10^th^ sample (83 ms intervals) after trial onset. With a Bonferroni corrected α level of 0.001, pupil diameter on trials with a remembered scene were significantly greater following a target tone than a distractor tone from 0.667 to 0.833 s after trial onset (Fig. 5a), *t’s*(41) > 3.877, *p’s* < 0.0004, *d’s* > 0.535. No other differences were significant at this α level. Without correction (α = 0.05), several effects were significant. First, pupil diameter on trials with a remembered scene was significantly greater following a target tone than a distractor tone from 0.583 to 1.75 s after trial onset (Fig. 5a), *t’s*(41) > 2.482, *p’s* < 0.017, *d’s* > 0.449. When the scene was forgotten, no differences between target and distractor trials were significant (though there was a trend at 0.5 s), *t’s*(41) < 1.861, *p’s* < 0.070. For target trials, pupil diameter was significantly greater from 0.500 to 0.667 s after trial onset when the scene was remembered rather than forgotten (Fig. 5b), *t’s*(41) > 2.018, *p’s* < 0.050, *d’s* > 0.117. No significant differences were observed for distractor-paired remembered and forgotten scenes, *t’s*(41) < 1.67, *p’s* > 0.102.

Similar results were found when residual pupil diameter was analyzed. For trials with a remembered scene, pupil diameter significantly differed on target and distractor trials from 0.583 to 1.75 s, *t’s*(41) > 2.482, *p’s* < 0.017, *d’s* > 0.449. For forgotten scenes, there was a trend toward a significant difference at 0.500 s, *t*(41) = 1.744, *p* = 0.089. From 0.500 to 0.667 s after trial onset, pupil diameter following target-paired remembered scenes showed a trend to being significantly greater than that for target-paired forgotten scenes, *t’s*(41) > 1.792, *p’s* < 0.080, *d’s* > 0.087. No significant differences were observed for distractor-paired remembered and forgotten scenes, *t’s*(41) < 1.671, *p’s* > 0.102. These analyses are consistent with the prediction that the difference in phasic pupil responses to remembered and forgotten scenes should be larger on target trials than on distractor trials.

Additional analyses examined whether other measures of pupil responses associated with encoding success (pupil constriction^55^), and task engagement^32,40^ (starting pupil diameter, pupil diameter before encoding related constrictions) differed across conditions. One prior study^55^ demonstrated that pupil constrictions 300–1000 ms after image onset were steeper when the images were subsequently remembered rather than forgotten. To facilitate comparisons with this study, pupil constrictions were defined as the slope of a linear regression line fit to the pupil time-course during the period 300–1000 ms after scene onset (*mid-latency slope*). Slopes were shallower for target trials than distractor trials, *F*(1, 41) = 4.27, *p* = 0.04. However, they did not differ for subsequently remembered vs. forgotten scenes, main effect, *F*(1, 41) = 0.030, *p* = 0.823, interaction, *F*(1, 41) = 0.039, *p* = 0.845. Analyses of starting pupil diameter and the intercepts from the linear regressions used to calculate the mid-latency slope also revealed no significant effects of encoding condition or subsequent memory, *F*(1, 41) < 3.216, *p* > 0.08.

Another analysis defined the time period of the mid-latency slope using the data itself. For each participant and condition, the maximum pupil diameter during the first second of the trial, and the minimum pupil diameter after that, were identified. A regression line was then fit to the data between the maximum and the minimum values (see Table 5 for descriptive statistics). Maximum values occurred significantly later on target trials than on distractor trials, *F*(1, 42.974) = 4.88, *p* = 0.032. In addition, there was a trend to a significant encoding condition by subsequent memory interaction effect on maximum pupil diameter, *F*(1, 82) = 3.639, *p* = 0.060. Post-hoc analyses indicated that for subsequently remembered scenes, maximum pupil diameter was greater following target tones than following distractor tones, *t*(41) = 2.252, *p* = 0.030, *d* = 0.273. This was not the case when scenes were subsequently forgotten, *t*(41) = −1.053, *p* = 0.298. For distractor trials, maximum pupil diameter was greater when the scene was subsequently forgotten than when it was subsequently remembered. This difference was marginally significant, *t*(41) = −1.962, *p* = 0.057, *d* = −0.350. For target trials, maximum pupil diameter did not differ significantly across trials that included subsequently remembered and forgotten scenes, *t*(41) = 0.648, *p* = 0.521. Encoding condition and subsequent memory did not have significant effects on the minimum pupil diameter, the time that it occurred, or the slope and intercept of the best fitting regression line (Table 6).

These analyses suggest that phasic pupil responses, operationalized as the area under the curve, were larger following the presentation of remembered target-paired scenes relative to the other conditions. These differences appear to reflect numerically smaller pupil diameters early in target trials with forgotten scenes relative to the other conditions (producing marginally significant differences in pupil diameter) combined with significantly slower decreases in pupil diameter following targets relative to distractors (indicated by the mid-latency slopes and significantly larger diameters for target trials following the initial peak). However, caution is warranted given the small number of trials and greater variability in the forgotten condition and the dependence of the slope and intercept analyses on how the windows are defined (Tables 4 and 5).

## Discussion

Most theories of selective attention account for dual-task interference between target detection and the processing of information that appears concurrently with the target, or shortly thereafter. However, the attentional boost effect shows dual-task facilitation when attention is directed to a target, not interference^16^. Because of this, it cannot be explained by accounts that were developed to explain interference over space and time. The dual-task interaction model proposes that the attentional boost effect reflects a temporal selection mechanism that briefly, but broadly enhances the processing of information presented at behaviorally relevant moments.

We asked whether the presentation of a target in a continuous dual-task increases pupil diameter while facilitating the encoding of concurrently presented scenes. The data suggest that it does: The presentation of a target rather than a distractor during encoding results in larger phasic pupil responses (Figs 3 and 4) and better subsequent memory (Fig. 2) for concurrently presented scenes. These differences were observed when the tone was equally likely to be a target or distractor, when targets required no overt motor response, when stimuli were presented at a relatively fast rate (one every two seconds), when multiple stimuli were attended, when multiple tasks were continuously performed, and for scenes with varying luminance.

Pupil responses to target and distractor tones also differed when the scene was subsequently remembered versus forgotten. Pupil diameter was larger for longer on trials that included a target and a subsequently remembered scene (Fig. 5). These results do not replicate previous findings that successful encoding is associated with faster decreases in pupil diameter during encoding^55,56^. Instead, they are more consistent with work associating larger pupil responses during encoding with subsequent, high confidence remember responses^27,54^, which also are more likely for target-paired images^8^. These results could at least partially reflect differences in pupil diameter at the beginning of target trials with subsequently forgotten scenes than with subsequently remembered scenes (suggested by a marginal trend for a difference in starting pupil diameter). However, the analyses were under-powered due to the small number of forgotten scenes (Table 3). Future research that reduces the imbalance across the subsequent memory conditions (perhaps by presenting the scenes fewer times) could therefore provide greater insight into the relationship between pupil diameter during encoding and later memory.

The results from this experiment are consistent with the dual-task interaction model, which proposes that the attentional boost effect arises from temporal selection^14^. They are also consistent with the potential role of a neurophysiological system thought to be involved in temporal selection^29^ and the attentional boost effect:^14^ the LC system. Oddball and vigilance tasks are often used to evoke phasic LC responses^23,28,38^. However, in previous studies phasic LC activity was not observed for a type of distractor that occurred as rarely as targets^34^, but was observed for discrimination tasks that included targets on most trials^61^. Phasic LC activity was more closely associated with the decision that a stimulus was a target and required a response, rather than the difficulty of distinguishing a target from a distractor, or the accuracy of that decision^28,34,61^. This suggests that phasic activity in the LC may be more closely associated with the categorization of a stimulus as a target, rather than the frequency with which a target appears relative to distractors, or the difficulty with which a target is discriminated from distractors. The attentional boost effect shows similar properties^8,14,17^.

The use of pupil diameter as a marker of LC activity has grown over the last several years, partially fueled by an interest in how task engagement, arousal, and motivation modulate perceptual and cognitive processing over time^32,45,62,63^. Recent reports used the inverse of the phasic pupillary response to trial onset as an index of tonic levels of LC activity and neural gain^50,64^. This measure is motivated by the finding that phasic changes in LC activity and pupil diameter are smaller when tonic levels of LC activity are high^28,38^. This work has demonstrated that smaller pupil responses to trial onsets are associated with the prioritization of task relevant and otherwise salient information (such as font shape when evaluating how easy a word is to read^64^), the precision with which perceptual information is represented in cortical areas^65^, and estimates of functional connectivity^50^. These findings support a relationship between the LC, neural gain, and the selection of behaviorally relevant stimuli.

Studies that use the inverse pupil response to study LC activity and neural gain also suggest the magnitude of phasic pupil responses to trial onsets may not provide a straightforward index of phasic changes in LC activity and neural gain. The observed differences in phasic pupil responses following target and distractor tones in our study could reflect larger phasic responses in the LC, or lower levels of tonic activity at the outset of the trial. However, starting pupil diameter was similar for target and distractor trials (Fig. 3c), and did not significantly differ during the encoding of remembered and forgotten scenes (Table 4, there were marginal effects in some related analyses, however). In addition, the effects of target detection on phasic pupil responses may not be eliminated by variability in tonic pupil diameter at the outset of the trial^44^. The expectation that larger phasic pupil responses reflect increased LC activity is further supported by neuroimaging work. Studies that examined differences in phasic pupil responses across conditions of an experimental manipulation (rather than as a naturally varying indicator of task-engagement or arousal over time), have found that greater phasic pupil responses are associated with greater LC activity^37,66^.

Despite growing evidence of LC involvement in task engagement and the processing of perceptual information, the functional and anatomical properties of the LC have yet to be fully characterized. Whereas previous work on the LC implied that its effects on processing were global, recent investigations demonstrate heterogeneity in the cell types, projections, receptors^67^, and functions of the LC^68^. The effects of LC activity on neural activity also may be influenced by the physiological environment surrounding a synapse, defined by the receptors and the presence of inhibitory inputs from other regions^68^. The LC also exhibits some modularity in its organization, with clusters of cells that project to different areas, such as the hippocampus and cortex^68^, with varying degrees of strength^69^. With moderate levels of input in the LC, activity of these modules may be separable, allowing it to influence processing in some systems more than others^70,71^. It will be important to test whether the phasic pupil responses observed here coincide with the modulation of regions involved in encoding scenes into memory (e.g., visual cortex and hippocampus) by the LC.

The relationship between the attentional boost effect and the phasic pupil responses reported here needs further exploration. Though recent data suggest that the LC plays a central role in modulating pupil diameter^39^, pupil diameter has also been associated with multiple neurophysiological systems involved in the regulation of bodily, affective, and cognitive states^32,45,49,53,72–74^. For example, pupil diameter increases following stimulation of the LC, acetylcholine neurons (potentially arising from the basal forebrain), and superior colliculus, a subcortical structure involved in selective attention, multi-sensory integration, and eye movements^23,25,28,38,75^. It also increases under dual-task demands and increases in memory load^47,49,76^. The mechanisms that produced differential pupil responses to targets during encoding therefore require additional research to isolate.

Future research should also investigate whether these effects are modulated by multi-sensory integration and orienting responses to locations in space. Both are tied to the superior colliculus, a central structure for generating eye movements, and are associated with phasic pupil responses^25^. These responses are larger for spatially co-localized auditory and visual signals than for stimuli presented in a single modality (multi-sensory integration^72^) and for spatially selected stimuli than for ignored stimuli^77^. Important questions are whether temporal selection modulates multi-sensory integration, and whether the effects of target detection on phasic pupil responses vary when stimuli are presented within the same modality rather than across modalities. Regardless, multi-sensory integration cannot account for the attentional boost effect, which occurs when all stimuli are presented within a single modality, and when stimuli are presented in separate spatial locations^7,14^.

Several aspects of the pupil responses observed in this study also require further investigation. The effect of targets on pupil diameter was evident through the end of the trial. This could reflect the slow nature of pupil responses to behaviorally relevant events, which can take several seconds to resolve^44,46^. For example, unexpected auditory stimuli produce phasic increases in LC activity (as well as inferior colliculus, superior colliculus, anterior cingulate, and posterior cingulate), followed by large, long lasting increases in pupil diameter that peak approximately 800 ms later^38^. In that study, pupil diameter had not returned to baseline by the end of the reporting period (1000 ms after tone onset, or after microstimulation of the LC). The extended nature of the pupil response could also reflect the interaction of phasic pupil responses with other aspects of the task, such as updating an internal counter for target tones. Alternatively, target detection may affect baseline levels pupil diameter, LC activity, and, by inference, effort^49,76,78^. Additional research is needed to test each of these possibilities.

### Conclusion

The attentional boost effect reflects enhanced processing, rather than interference, of items that are presented with concurrently behaviorally relevant target stimuli. Because these effects are the opposite of what is typically observed in attention tasks, they are unlikely to result from mechanisms that bias perceptual processing toward behaviorally relevant items, locations, or features (as in biased competition^79^). Rather, better memory for target-paired items and contexts is likely to reflect the engagement of a mechanism that broadly, but briefly, enhances the processing of information presented at behaviorally relevant moments. By demonstrating that targets are associated with larger phasic pupil responses than distractors, particularly when they are remembered, this study provides further evidence that temporal selection is a potential source of these enhancements.

## Methods

### Participants

Forty-three participants (24 female, 19 male, 18–33 years old, mean age 21.15 years) completed the experiment after providing informed consent. One participant was excluded due to poor performance on the counting task (<60% accuracy). With a sample size of 42 participants, this experiment had power (1-*β*) of 0.8 to detect an effect of *d* = 0.443 in a two-tailed, matched samples *t*-test with *α* = 0.05 (calculated with G*Power, Faul, *et al*., 2007). All procedures were approved by the University of Minnesota IRB and were performed in accordance with the standards of the Declaration of Helsinki.

### Materials and Equipment

All stimuli were presented on a 17-inch CRT color monitor (1024 × 768 pixels, 75 Hz vertical refresh rate) or with computer speakers using MATLAB (Mathworks) and Psychtoolbox^80,81^. Participants rested their heads on a chin rest 86 cm from the monitor, and adjusted the height of their chair to ensure their comfort.

Point of regard (x and y coordinates) and pupil diameter were measured with an ISCAN ETL-300 infrared eye tracking system (512 × 512 pixel spatial resolution, 120 Hz sample rate), controlled by a PC computer running the ISCAN DQW software.

A set of 224 full color images of outdoor and indoor scenes (256 × 256 pixels) was acquired from a collection used in previous studies^4^. For each participant, 112 of the scenes were randomly assigned to be presented during the encoding and counting task and recognition test (*old scenes*) and 112 were randomly assigned to appear as foils during the recognition test (*new scenes*). For each participant the old scenes were randomly and evenly assigned to either always appear with a target tone (*target-paired scenes*) or to always appear with a distractor tone (*distractor-paired scenes*) during encoding, resulting in 56 scenes per condition. A mask was created for each scene by dividing it into 256 squares (16 × 16 pixels), and then shuffling their locations. An additional 48 scenes were used for the encoding and counting task practice. Scene luminance was defined as the mean pixel value (0–255) of an image after it was converted to grayscale (using MATLAB’s rgb2gray). Luminance varied across scenes (range = 32.13–175.04; M = 120.60; SD = 24.0). This luminance value captures the relative luminance of the scenes, rather than the absolute amount of light emitted by the display, which we were unable to directly measure. However, the luminance values obtained from the images were strongly correlated with a range of gamma corrected luminance values, ranging from *r* = 0.996 with gamma = 1.5 to *r* = 0.959 with gamma = 3.0. In addition, gamma correcting the luminance values did not substantively change the results (with a typical gamma correction of 2.5, the correlation between luminance and pupil responses on distractor trials changed from −0.59 to −0.60; the correlation did not change for target trials).

### Task Design and Procedure

Participants completed the encoding and counting task while pupil diameter and gaze location were recorded with an eye tracker. Participants were instructed to maintain fixation on a central fixation cross and to minimize blinks. After the encoding and counting task, they completed a recognition memory test on the scenes without eye tracking. At the end of the scene recognition task, participants also completed working memory span tests as pilot data for a larger project. These were unrelated to the focus of the present study.

### Encoding and Counting Task

Participants were asked to perform two tasks at once (Fig. 1a). For the *counting task* they monitored a series of individually presented high (650 Hz) and low (350 Hz) tones (100 ms duration; 1900 ms inter-tone-interval). They counted the number of times they heard a *target* tone, which was defined by pitch (high for half of the participants and low for the other half of the participants). They did not count the number of tones in the other pitch (*distractor*). For the *encoding task* participants memorized a series of individually presented scenes (5.8° × 5.8°) presented at the center of the computer screen over a gray background. Each scene was presented for 500 ms, and then masked with a scrambled version of itself for 1500 ms. The scenes and tones onset at the same time, forming a single *trial* lasting 2000 ms. Each scene was assigned to always appear with either a target or distractor tone, resulting in two, equally frequent scene *encoding conditions* (target paired or distractor paired scenes). A red fixation cross (0.5° × 0.5°) appeared in the center of the screen, overlaid on the other visual stimuli, throughout the trial. The mask from one trial was immediately followed by the scene for the next trial (0 ms inter-trial interval). Each scene was presented 6 times (each time in the same encoding condition), resulting in a total of 672 trials. Half of the scenes were always paired with a target tone, and the other half were always paired with a distractor tone. Trials were pseudo-randomly ordered such that there could be no more than two target or distractor tones in a row, and all scenes were presented once per repetition. This ensured that scene presentations were distributed throughout the task.

The task paused after 6–10 target trials for participants to report their current count of the number of target tones they had heard (Fig. 1b). The number of distractor tones during the same period was not controlled, but because of the equal frequency of tones it was roughly equivalent to the number of target tones. When the count probe display appeared, participants pressed a button corresponding to the number of target tones they had counted since the last probe. Following their response, participants were given feedback and an opportunity to rest before restarting the task. The task paused for calibration and validation of the eye tracker every 224 trials (14 count probes; 112 targets and 112 distractors).

Participants completed a practice with 48 practice scenes, each presented once, before beginning the encoding and counting task.

### Image Recognition Memory Test

After completing the encoding and counting task, participants performed a two-alternative forced choice recognition test on the scenes. On each trial they viewed one old scene and one new scene (5.8° × 5.8°). The side on which the old scene appeared was counterbalanced across trials. Participants pressed a key to pick the old scene and were instructed to be as accurate as possible.

### Eye Data Recording and Processing

Prior to beginning the encoding and counting task the experimenter adjusted the eye tracker to acquire a stable image of the left eye and to adjust the thresholds used to identify pupil and corneal reflectance. The eye tracker was then calibrated to the participant’s eye using a 5-point calibration technique (repeated every 224 trials). The horizontal and vertical coordinates of the participant’s point of regard and their pupil diameter were recorded.

Blinks were detected automatically by the eye tracker, and additional blink and artifact filtering was performed in MATLAB. A data point was excluded if it occurred within 10 frames of a data point identified by the eye tracker as a blink, if it was more than 3.5 standard deviations above the mean pupil diameter for that particular trial, or if it differed by more than 5 arbitrary units from the previous data point (indicating nonphysiological noise). This latter cutoff was conservative: across all participants and trials, the mean change in pupil diameter across samples (8.3 ms intervals) was <0.001 arbitrary units. Linear interpolation was used to replace all data lost to blinks. However, trials on which nonphysiological noise was detected were excluded from the analyses. After applying this algorithm, 7.8% of all trials, 65.6% of which were target trials, were excluded.

Starting pupil diameter and the phasic pupil response were estimated for the first 1.75 s of each trial (0 = tone onset). Pupil diameter was normalized (z-scored) by subtracting the participant’s mean diameter and dividing by their standard deviation. Because pupil dilation following spiking activity of LC neurons is typically delayed by more than 200 ms^38,82^, the first 100 ms of the trial were averaged to obtain *starting pupil diameter*. *Phasic pupil response* was quantified by calculating the area under the curve, using the trapz function in the caTools R package^83^.

Additional analyses characterized pupil responses following the initial peak in pupil diameter following tone onset. One analysis replicated an earlier report by fitting a linear model to pupil diameter during the 300–1000 ms time period following pupil response^55^. This time period was chosen to allow comparisons across studies. The linear model included an *intercept*, a *slope* characterizing the rate of change in pupil diameter over time, and an error term. One model was fit for each combination of participant, encoding condition, and subsequent memory (whether a scene was later accurately recognized or not). A second analysis also sought to characterize the post-peak decrease in pupil diameter. However, this analysis determined the time period over which the decrease occurred for each combination of participant, encoding condition, and subsequent memory accuracy. The beginning of the time period was defined as the time point at which pupil diameter was at its maximum during the first second of the trial. The end of the time period was defined as the time point at which the pupil diameter was at its minimum after the maximum occurred. One linear model including intercept and slope terms was then fit to the data for each participant and condition as before.

Gaze position changes were examined to determine whether participants followed instructions to fixate. As with pupil diameter, trials on which nonphysiological noise produced an abnormally large change in gaze position, quantified as sample to sample distance (greater than 3 units, where the mean distance across all participants and samples was 0.027) were removed from the analysis. Gaze position was then down-sampled to 12 samples per second, converted to distance, and averaged within participant. Paired t-tests indicated that mean sample-to-sample distances in gaze location did not significantly differ across target and distractor trials, distractor M = 0.632, SD = 0.194; target M = 0.642, SD = 0.217, *t*(41) = 1.542, *p* = 0.131. Values are in units reported by the eye tracker, but can be converted to visual degrees by dividing by 23.7.

## Data Availability

The data collected and analyzed for the current study are available from the corresponding author on reasonable request.
